# A Prospective Study on Rapidly Declining SARS‐CoV‐2 IgG Antibodies Within One to Three Months of Testing IgG Positive: Can It Lead to Potential Reinfections?

**DOI:** 10.7759/cureus.11845

**Published:** 2020-12-02

**Authors:** Deb Sanjay Nag, Rajan Chaudhry, Minakshi Mishra, Sudhir Rai, Minakshi Gupta

**Affiliations:** 1 Anaesthesiology, Tata Main Hospital, Jamshedpur, IND; 2 Medical Services, Tata Main Hospital, Jamshedpur, IND; 3 Pathology, Tata Main Hospital, Jamshedpur, IND; 4 Microbiology, Tata Main Hospital, Jamshedpur, IND

**Keywords:** antibody response, covid-19, immunoglobulin g

## Abstract

Background

COVID-19 immunoglobulin G (IgG) antibodies have been considered to provide protective immunity and its immunoassays have been widely used for serosurveillance. In our serosurveillance on an industrial workforce of randomly selected 3296 subjects, COVID-19 IgG antibody positivity was reported in 7.37% (243) subjects. However, when 30 days later, eight of the 243 COVID-19 IgG antibody-positive individuals complained of symptoms suggestive of COVID-19 infection and were confirmed as COVID-19 infection by reverse transcription-polymerase chain reaction (RT-PCR), their COVID-19 IgG antibodies were retested. Seven of the eight previously IgG positive individuals had lost their protective antibodies.

Methods

Subsequently, a prospective clinical trial was planned by repeating the test for IgG antibodies on the remaining earlier positive 235 individuals at 45-65 days after their initial test. Only 201 of the 235 individuals consented and participated in the non-randomized single-arm observational trial.

Results

Only 28.36% (57/201) retained their IgG antibodies and 70.15% (141/201) had lost their IgG antibodies. Three cases reported equivocal results on retesting.

Conclusions

Our findings show that the protective COVID-19 IgG antibodies rapidly decline over one to three months. Further studies are needed with a quantitative assay over a period with neutralizing antibodies to establish if its decay can potentially lead to reinfections. Rapidly decaying protective IgG antibodies would impact herd immunity and vaccine durability. It is critical for the potential vaccines to generate both protective T- and B-cell immune responses in a sustained manner.

## Introduction

The COVID-19 pandemic has engulfed the entire globe with over 40.1 million cases and 1.1 million deaths being reported worldwide. Retrospective serosurveillance is often used to screen for unidentified previous or mild infection with severe acute respiratory syndrome coronavirus 2 (SARS-CoV-2) and serves as an important tool to “screen for and interrupt undetected chains of disease transmission” [1].

Various immunoassays of immunoglobulin G (IgG) antibodies have been developed and are being widely used across the world for SARS-CoV2. While reverse transcription-polymerase chain reaction (RT-PCR) remains the gold standard for identifying viral ribonucleic acid (RNA), the viral load reduces drastically in nine to ten days after infection and cannot be used for retrospective surveillance [1]. Serology testing helps us in retrospectively determining previous SARS-CoV-2 infections in people who have not been tested earlier by an RT-PCR. While most detect specific antibodies against the spike/receptor-binding-domain or nucleocapsid [2], limited commercial availability of approved kits to assess neutralizing antibodies against the virus [3] has restricted the wider use for accurate testing of neutralizing antibody titers.

It was earlier estimated that the pathogenicity of SARS-CoV-2 is “like SARS-CoV in some ways” [4]. However, recent studies have expressed doubts on the longevity and the “protective immunity” provided by the SARS‐CoV‐2 IgG antibodies [5]. While some initial studies have shown that 40% of asymptomatic individuals and 12.9% of symptomatic individuals become seronegative for IgG antibodies in the “early convalescent phase” [6], there are other isolated reports of rapid decay of IgG antibodies in persons with a mild infection [7]. 

Background

As a serosurveillance measure, an organizational protocol was designed and 3296 asymptomatic employees between 21 and 60 years of age of either sex in an industrial workforce at Jamshedpur (India) were tested for SARS‐CoV‐2 IgG antibodies specific for the spike subunit antigen by the ErbaLisa COVID-19 (Erba Corporate Services, United Kingdom) between June 28 and July 15, 2020. Based on validation conducted in the USA and Italy, its diagnostic sensitivity and specificity have been reported to be 98.3% and 98.1% [8]. All individuals participating in the serosurveillance gave written informed consent to participate in the program. The findings of the serosurveillance for SARS‐CoV‐2 IgG antibodies is depicted in Table 1. Two-hundred forty-three (243) of the tested employees were positive for SARS‐CoV‐2 IgG antibodies, showing an overall positivity of 7.37%. Based on the manufacturer guidelines, an optical density (OD) ratio by enzyme-linked immunosorbent assay (ELISA) above 1.1 was considered positive, OD ratio between 0.9 and 1.1 was considered equivocal, and OD ratio below 0.9 was considered negative for SARS‐CoV‐2 IgG antibodies.

All those who were reported as SARS‐CoV‐2 IgG antibody positive or equivocal were personally interviewed after the test results were available. No individual reported any symptoms suggestive of COVID-19 in the preceding two to three months.

Subsequently, a cohort of eight employees who tested positive for SARS‐CoV‐2 IgG antibodies during the serosurveillance reported symptoms of influenza-like illness (ILI) fever, cough, and headache after about 30 days of their initial testing as SARS‐CoV‐2 IgG antibodies positive. Their nasopharyngeal swab samples were taken and an RT-PCR assay was done by the STANDARD M nCoV Real-Time Detection kit (SD Biosensor, Republic of Korea). All eight symptomatic employees tested positive for SARS-CoV-2. As per the manufacturer’s instructions, a cycle threshold (Ct) value below 36 for the ORF1ab (RdRp) gene and the E gene was considered SARS-CoV2 positive. Seven of these eight earlier SARS-CoV-2 IgG positive individuals and now RT-PCR positive for SARS-CoV-2 were now negative for SARS-CoV-2 IgG antibodies. All eight patients were managed conservatively and had an uneventful recovery.

## Materials and methods

Table 1 shows the findings of the serosurveillance for SARS‐CoV‐2 IgG antibodies.

**Table 1:** Findings of the serosurveillance for SARS‐CoV‐2 IgG antibodies SARS‐CoV‐2: severe acute respiratory syndrome coronavirus 2; IgG: immunoglobulin G

	Positive	Negative	Equivocal
Numbers	243	3007	46
Percentage	7.37%	91.23%	1.40%

The loss of the protective antibodies led the team to reassess its presence in all cases who tested positive for SARS‐CoV‐2 IgG between June 28 and July 15, 2020. In view of the unexpected findings of having an RT-PCR positive infection in employees who were SARS‐CoV‐2 IgG antibody positive less than 40 days ago, based on availability, a non-randomized single-arm observational trial on the remaining 235 cases using the SARS-CoV-2 IgG assay by the Access SARS-CoV-2 IgG assay (Beckman Coulter, Brea, California) was planned. The Access SARS-CoV-2 IgG assay detects antibodies to the receptor-binding domain (RBD) of the spike protein. Based on the manufacturer guidelines, automated chemiluminescent immunoassay (CLIA) with the Access SARS-CoV-2 IgG assays, a signal-to-cut-off (S/CO) ratio above 1.0 was considered positive, a S/CO ratio between 0.8 and 1.0 was considered equivocal, and OD ratio below 0.8 was considered negative for SARS‐CoV‐2 IgG antibodies. The Access SARS-CoV-2 IgG assay is reported to have 100% sensitivity and 99.8% specificity [9].

**Figure 1:**
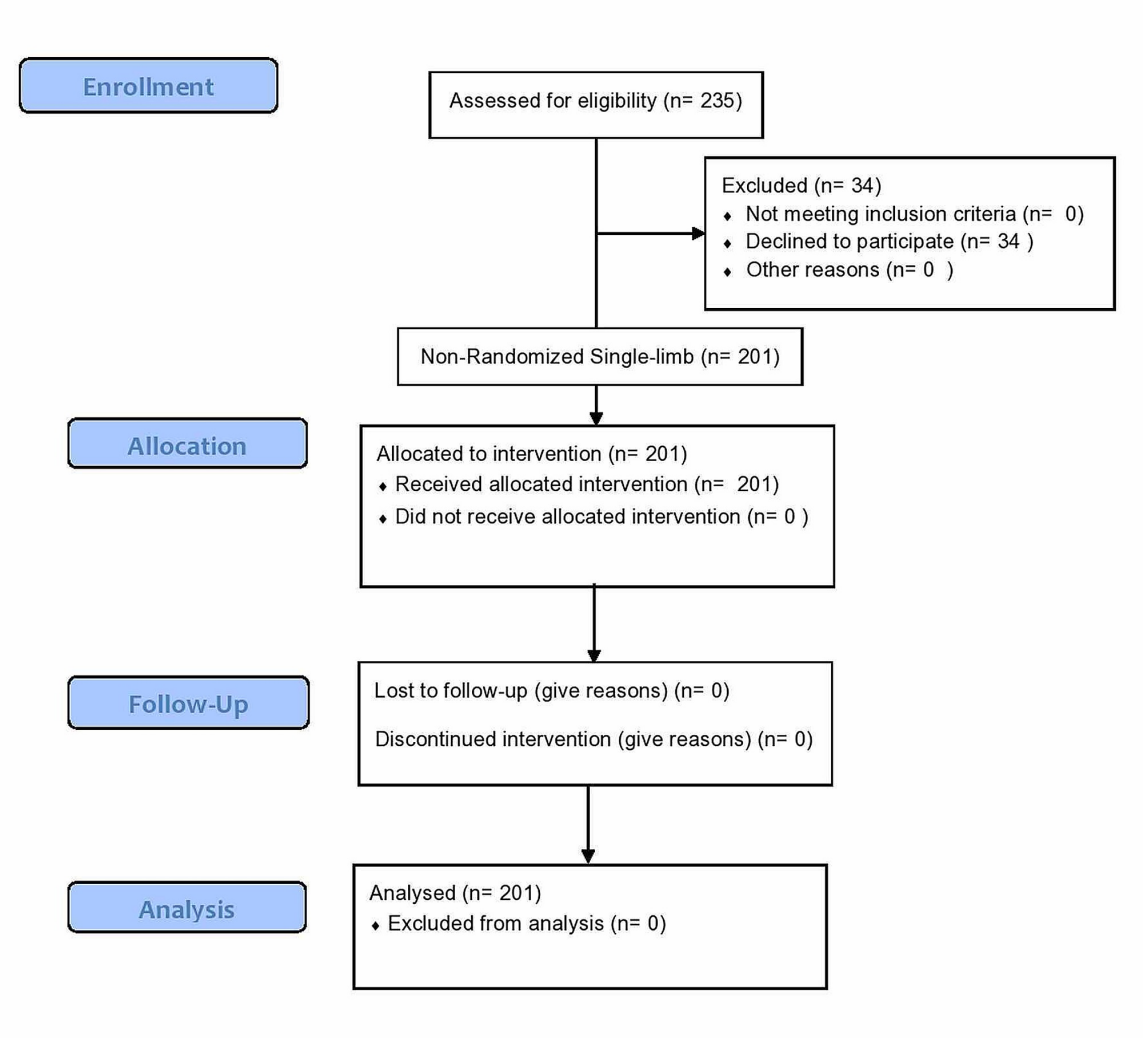
CONSORT flow diagram

The study aimed to find out the percentage of cases with SARS-CoV-2 IgG antibodies retaining the antibodies 45-65 days of initially testing positive. All cases that had initially tested positive for SARS-CoV-2 IgG antibodies between June 28 and July 15, 2020, were included in the study. The CONSORT flow diagram is depicted in Figure 1. 

Trial Registration

The observational study is retrospectively registered with ClinicalTrials.gov on October 27, 2020 (Registration identification No. NCT04605952) available at https://clinicaltrials.gov/ct2/show/NCT04605952.

## Results

Only 201 of the 235 could be recruited for a repeat SARS-CoV-2 IgG assay 49-63 days after they had initially tested positive. The results are tabulated in Table 2. Only 28.36% of the earlier SARS‐CoV‐2 IgG positive cases retained their protective antibodies. The findings of the repeat testing for SARS‐CoV‐2 IgG antibodies is depicted in Table 3. The comparison of the ELISA OD ratio range on initial testing between June 28 to July 15, 2020, and subsequent CLIA S/CO ratio range between August 27 and August 29, 2020, is depicted in Table 4.

**Table 2:** Results of SARS-CoV-2 IgG antibodies and RT-PCR SARS‐CoV‐2: severe acute respiratory syndrome coronavirus 2; IgG: immunoglobulin G; OD: optical density; ELISA: enzyme-linked immunosorbent assay; Ct: cycle threshold; S/CO: signal-to-cut-off; CLIA: chemiluminescent immunoassay

	OD Ratio of SARS‐CoV‐2 IgG antibodies by ELISA on July 8, 2020	RT-PCR ORF1ab (RdRp) gene Ct values on August 11, 2020	RT-PCR E gene Ct values on August 11, 2020	S/CO Ratio of SARS‐CoV‐2 IgG antibodies by CLIA on August 14, 2020
Case 1	3.297	14	15	0.32
Case 2	1.385	17	19	0.6
Case 3	1.39	23	24	2.03
Case 4	1.69	13	14	0.02
Case 5	1.432	11	13	0.03
Case 6	1.563	14	16	0.07
Case 7	2.127	11	13	0.61
Case 8	2.058	20	21	0.07

**Table 3:** Findings of the repeat testing for SARS‐CoV‐2 IgG antibodies in previously positive individuals SARS‐CoV‐2: severe acute respiratory syndrome coronavirus 2; IgG: immunoglobulin G

	Positive	Negative	Equivocal
Numbers	57	141	3
Percentage	28.36%	70.15%	1.49%

**Table 4:** Comparison of ELISA OD ratio range and CLIA S/CO ratio range OD: optical density; ELISA: enzyme-linked immunosorbent assay; S/CO: signal-to-cut-off; CLIA: chemiluminescent immunoassay

	CLIA S/CO Ratio Range (August 27 to August 29, 2020)								
ELISA OD Ratio Range (June 28 to July 15, 2020)	<0.8	0.8 to 1	1 to 2	2 to 3	3 to 4	4 to 5	5 to 10	>=10	Grand Total
1 to 2	102	2	6	4	1	1	12	10	138
2 to 3	26	1	4	4	2	2	1	6	46
3 to 4	11	0	0	0	0	0	1	2	14
4 to 5	2	0	0	0	0	0	0	0	2
>=5	0	0	0	1	0	0	0	0	1
Grand Total	141	3	10	9	3	3	14	18	201

## Discussion

Apart for screening for potential donors for convalescent plasma in patients who have recovered from COVID-19, screening for SARS‐CoV‐2 IgG antibodies would help establish the true prevalence of the disease in the community. Given the wide variability in the incidence of asymptomatic infection ranging from 4%-80% [10], it could potentially identify the actual number of infected in a defined population. However, our observation of 70.15% of the individuals losing their protective antibodies in 49-63 days shows that we may not be ever able to accurately predict the prevalence of the infection. While our findings are similar to the observations of Long QX [6] and Ibarrondo FJ [7], their observations were based on only 37 and 34 participants. Our sample size of 201 cases is possibly the largest sample in which the decay of SARS‐CoV‐2 IgG antibodies over one to three months have been reported to date. While our study observed the effect of loss of protective IgG antibodies in asymptomatic individuals, further studies need to be conducted to assess its impact on symptomatic individuals.

While there are isolated case series reporting SARS‐CoV‐2 reinfection [11], given the high sensitivity and specificity of the ErbaLisa COVID-19 Enzyme Immunoassay kits (98.3% and 98.1%), seven of the eight RT-PCR confirmed cases in the initial findings can be possibly considered as reinfections. However, in the absence of RT-PCR confirmation of the previous infection leading to the initial SARS‐CoV‐2 IgG antibody-positive results, reinfection cannot be confirmed. Besides, in the absence of neutralizing antibody tests, semi-quantitative assays of SARS‐CoV‐2 IgG antibody may only indicate prior exposure to the SARS‐CoV‐2 infection and “does not equate to protective immunity” [10]. Standard plaque reduction neutralization tests (PRNTs) or the currently investigational neutralizing impact on pseudotyped vesicular stomatitis virus (VSV) expressing different SARS-CoV-2 surface antigens would be needed to establish humoral immunity [10]. While in 7 of the 8 cases, it was established that decay of the protective IgG antibodies made the individuals susceptible to subsequent SARS-CoV-2 infection, only one of the cases remained positive for SARS‐CoV‐2 IgG antibodies. The only case that was positive for SARS‐CoV‐2 IgG antibodies and RT-PCR positive for SARS‐CoV‐2 can possibly be explained by positive signals from dead viruses or fragmented viral genes without actual viral replications or infectivity [12].

The rapid decay of the SARS‐CoV‐2 IgG antibody in one to three months could be established through our findings. This would not only impact our current understanding on monitoring the potential vaccine recipients, but it would also make it critical for the potential vaccines to generate both “protective T- and B-cell immune responses” in a sustained manner [13]. A mild infection may not provide long-lasting humoral immunity. Given the fact that the majority of COVID-9 infections are mild, our findings show that rapidly decaying protective IgG antibodies would impact herd immunity and vaccine durability [7]. Further studies on quantitative assays of neutralizing antibodies would define the duration of protection provided by the SARS‐CoV‐2 IgG antibodies.

Limitations of the study

While assessing the SARS‐CoV‐2 IgG antibody through ELISA initially and by CLIA on follow-up can potentially give rise to confounding factors, current evidence shows that any of these assays can “effectively detect SARS-CoV-2 antibodies.” Besides, other studies for hepatitis B surface antigen [14] and Mycoplasma pneumoniae [15] show that while there may be minor discrepancies in quantitative measurements, there is good analytical agreement and high concordance between CLIA and ELISA.

While all the initially IgG antibody-positive individuals were personally interviewed about any symptoms suggestive of COVID-19 in the preceding two to three months, their response can be potentially susceptible to recall bias.

## Conclusions

Our findings show that SARS‐CoV‐2 IgG antibodies rapidly decay over one to three months and can potentially result in reinfections. These findings would impact our understanding of herd immunity and the monitoring of potential vaccine recipients.
